# Plasma cell myeloma with *RAS/BRAF* mutations is frequently associated with a complex karyotype, advanced stage disease, and poorer prognosis

**DOI:** 10.1002/cam4.6103

**Published:** 2023-05-22

**Authors:** Nianyi Li, Pei Lin, Zhuang Zuo, M. James You, Wen Shuai, Robert Orlowski, Elisabet E. Manasanch, Shaoying Li, Jie Xu, Sofia Garces, Fatima Zahra Jelloul, Zhenya Tang, Wei Wang, L. Jeffrey Medeiros, C. Cameron Yin

**Affiliations:** ^1^ Department of Hematopathology University of Texas MD Anderson Cancer Center Texas Houston USA; ^2^ Department of Lymphoma/Myeloma University of Texas MD Anderson Cancer Center Texas Houston USA

**Keywords:** *BRAF*, *KRAS*, *NRAS*, Plasma cell myeloma, poor prognosis

## Abstract

**BACKGROUND:**

Mutations in the RAS‐MAPK pathway, such as *KRAS*, *NRAS*, and *BRAF*, are known as high‐risk factors associated with poor prognosis in patients with various cancers, but studies in myeloma have yielded mixed results.

**METHODS:**

We describe the clinicopathologic, cytogenetic, molecular features, and outcomes of 68 patients with *RAS/BRAF*‐mutated myeloma, and compare with 79 patients without any mutations.

**RESULTS:**

We show that *KRAS*, *NRAS*, and *BRAF* were mutated in 16%, 11%, and 5% of cases, respectively. *RAS/BRAF*‐mutated patients had lower hemoglobin and platelet counts, higher levels of serum lactate dehydrogenase and calcium, higher percentage of bone marrow plasma cells, and more advanced R‐ISS stage. *RAS/BRAF* mutations were associated with complex karyotype and gain/amplification of *CKS1B*. The median overall survival and progression‐free survival were significantly shorter for *RAS/BRAF*‐mutated patients (69.0 vs. 220.7 months, *p* = 0.0023 and 46.0 vs. 60.6 months, *p* = 0.0311, respectively). Univariate analysis revealed that *KRAS* mutation, *NRAS* mutation, lower hemoglobin, elevated lactate dehydrogenase, higher R‐ISS stage, complex karyotype, gain/amplification of *CKS1B*, monosomy 13/*RB1* deletion and lack of autologous stem cell transplantation were associated with poorer prognosis. Multivariate analysis showed that *KRAS* mutation, lower hemoglobin level, higher level of serum calcium, higher ISS stage, and lack of autologous stem cell transplantation predict inferior outcome.

**CONCLUSIONS:**

*RAS/BRAF* mutations occur in 30%–40% of myeloma cases and are associated with higher tumor burden, higher R‐ISS stage, complex karyotype, and shorter overall survival and progression‐free survival. These findings support testing for *RAS/BRAF* mutations in myeloma patients and underscore the potential therapeutic benefits of RAS/BRAF inhibitors.

## Graphical abstract

*RAS/BRAF* mutations occur in 30‐40% of myeloma cases and are associated with a higher tumor burden, higher R‐ISS stage, complex karyotype, and shorter overall survival and progression‐free survival. These findings support testing for *RAS/BRAF* mutations in myeloma patients and underscore the potential therapeutic benefits of RAS/BRAF inhibitors.

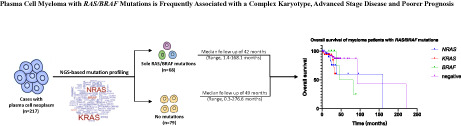

## 1 INTRODUCTION

Plasma cell myeloma is a neoplasm of clonal plasma cells that often begins as monoclonal gammopathy of undetermined significance (MGUS), progresses to symptomatic disease, and eventually becomes refractory to therapy.^1^, ^2^, ^3^ Despite recent advances in treatment strategies including proteasome inhibitors, immunomodulatory drugs, and targeted monoclonal antibodies that have prolonged the overall survival (OS) of patients with myeloma, this neoplasm is still considered incurable with a poor clinical outcome for most patients.^4^ Tumor heterogeneity is a key factor influencing the variation in clinical course and survival of myeloma patients.^1^, ^5^ Recently, our understanding of the molecular pathogenesis of myeloma has greatly increased, accompanied by the development of molecular diagnostic methods now available to predict clinical outcome and improve the treatment of myeloma patients.^6^, ^7^ The Revised International Staging System (R‐ISS) developed in 2015 incorporated chromosomal abnormalities detected by fluorescence in situ hybridization (FISH) as well as the serum lactate dehydrogenase (LDH) level into the preexisting International Staging System (ISS).^8^ More recently, next‐generation sequencing (NGS) studies using whole‐genome and whole‐exome approaches have identified recurrent gene mutations of prognostic importance in myeloma.^9^, ^10^

Others have shown that myeloma is driven by mutations in the RAS signaling pathway, and that *KRAS/NRAS* mutations are present in 20%–50% and 45%–80% of newly diagnosed and relapsed/refractory myeloma cases, respectively.^9^, ^11^, ^12^, ^13^ *KRAS/NRAS* mutations have been associated with malignant transformation and a more aggressive clinical course.^14^ Activating *BRAF* mutations have been identified in 2%–5% of myeloma patients and have therapeutic relevance.^15^, ^16^, ^17^ However, the prognostic relevance of mutations in genes of RAS/BRAF pathway in myeloma remain unclear with contradictory published results, especially in the era of novel therapeutic agents. In most studies, patients with mutations in *KRAS*, but not *NRAS*, have had a higher bone marrow (BM) tumor burden and shorter survival.^18^, ^19^, ^20^, ^21^ Others have reported that *NRAS* mutations, but not *KRAS* mutations, negatively influenced the clinical outcome of patients treated with bortezomib.^9^ On the contrary, Gebauer et al. reported *RAS* mutations appeared to be associated with longer OS, progression‐free survival, and post‐relapse survival,^22^ whereas others reported no prognostic impact at all.^13^

In this study, our goal is to assess the clinical implications of *KRAS/NRAS/BRAF* mutations in myeloma patients, as these mutations have potential implications for targeted therapy. We therefore systematically analyzed the clinicopathologic, cytogenetic, and molecular genetic features as well as OS in a large group of myeloma patients with *KRAS/NRAS/BRAF* mutations.

## 2 MATERIALS AND METHODS

### 2.1 Case selection

We searched the database of our institution from November 1, 2014 to December 31, 2020 for cases of myeloma that underwent NGS‐based mutation profiling. The study group included patients who only had mutations in *KRAS*, *NRAS* or *BRAF*, without any other concurrent mutations to avoid confounding factors. A group of age, gender, and stage‐matched patients from the same study period with wild type tumors were selected as control. Both the study group and the control group contained newly diagnosed patients and relapsed/refractory patients. The clinical and laboratory data were obtained by review of medical records. The stages of disease were assessed using the ISS and R‐ISS systems. The study was conducted according to an Institutional Review Board‐approved protocol and in accord with the Declaration of Helsinki.

### 2.2 Morphologic evaluation.

We reviewed Wright‐Giemsa‐stained BM aspirate smears/touch imprints, and hematoxylin–eosin‐stained clot and core biopsy specimens. Differential counts on 500 cells were performed manually on BM smears/touch imprints.

### 2.3 Flow cytometry immunophenotyping

Multicolor flow cytometry immunophenotyping was performed on BM aspirates using a FACScan instrument (Becton‐Dickinson) as described previously.^23^ In all cases, 8‐color analysis was performed and plasma cells were identified by bright CD38/CD138 expression. The panel of other monoclonal antibodies included reagents specific for CD19, CD20, CD27, CD28, CD56, CD81, CD117, and cytoplasmic immunoglobulin kappa and lambda light chains (Becton‐Dickinson). An isotype control was used with each antibody.

### 2.4 Cytogenetic analysis

Conventional cytogenetic analysis was performed on metaphase cells prepared from BM aspirates cultured for 24–48 h without mitogens, using standard techniques. Twenty Giemsa‐banded metaphases were analyzed, and the results were reported using the International System for Human Cytogenetic Nomenclature, 2020.

FISH analyses were performed on interphase nuclei using a panel of probes for the detection of deletion of 1p32.3 (*CDKN2C*), gain/amplification of 1q21 (*CKS1B*), t(4;14)(*FGFR3*::*IGH*), t(11;14)(*CCND1*::*IGH*), deletion of *RB1* and chromosome 13 aneuploidy (13q14/13q34), t(14;16)(*IGH::MAF*), and deletion of 17p13.1 (*TP53*) or monosomy 17 according to the manufacturer's instructions (Vysis/Abbott Laboratories). A total of 200 nuclei were analyzed and a percentage was calculated by dividing the number of abnormal nuclei by total nuclei counted for each probe.

### 2.5 Molecular analysis

Upon presentation at our hospital, genomic DNA extracted from BM aspirates was amplified by polymerase chain reaction (PCR)‐based methods and subjected to mutation analysis. We used panels of either 53 (*n* = 47) or 81 (*n* = 21) genes that are commonly mutated in hematopoietic neoplasms and performed NGS using an Illumina MiSeq NGS platform (Illumina Inc.), as described previously.^24^ The panel of 53 genes included *ABL1*, *AKT1*, *ALK*, *APC*, *ATM*, *BRAF*, *CDH1*, *CDKN2A*, *CSF1R*, *CTNNB1*, *DNMT3A*, *EGFR*, *ERBB2*, *ERBB4*, *EZH2*, *PBXW7*, *FGFR1*, *FGFR2*, *FGFR3*, *FLT3*, *GNA11*, *GNAQ*, *GNAS*, *HNR1A*, *HRAS*, *IDH1*, *IDH2*, *JAK2*, *JAK3*, *KDR*, *KIT*, *KLHL6*, *KRAS*, *MET*, *MLH1*, *MPL*, *NOTCH1*, *NPM1*, *NRAS*, *PDGFRA*, *PIK3CA*, *PTEN*, *PEPN11*, *RB1*, *RET*, *SMAD4*, *SMARCB1*, *SMO*, *SRC*, *STK11*, *TP53*, *VHL*, and *XPO1*. The panel of 81 genes included *ANKRD26*, *ASXL1*, *ASXL2*, *BCOR*, *BCORL1*, *BRAF*, *BRINP3*, *CALR*, *CBL*, *CBLB*, *CBLC*, *CEBPA*, *CREBBP*, *CRLF2*, *CSF3R*, *CUX1*, *DDX41*, *DNMT3A*, *EED*, *ELANE*, *ETNK1*, *ETV6*, *EZH2*, *FBXW7*, *FLT3*, *GATA1*, *GATA2*, *GFI1*, *GNAS*, *HNRNPK*, *HRAS*, *IDH1*, *IDH2*, *IKZF1*, *IL2RG*, *IL7R*, *JAK1*, *JAK2*, *JAK3*, *KDM6A*, *KIT*, *KMT2A*, *KRAS*, *MAP2K1*, *MPL*, *NF1*, *NOTCH1*, *NPM1*, *NRAS*, *PAX5*, *PHF6*, *PIGA*, *PML*, *PRPF40B*, *PTEN*, *PTPN11*, *RAD21*, *RARA*, *RUNX1*, *SETBP1*, *SF1*, *SF3A1*, *SF3B1*, *SH2B3*, *SMC1A*, SMC3, *SRSF2*, *STAG1*, *STAG2*, STAT3, *STAT5A*, STAT5B, *SUZ12*, *TERC*, *TERT*, *TET2*, *TP53*, *U2AF1*, *U2AF2*, *WT1*, and *ZRSR2*. The detection sensitivity was 1%.

### 2.6 Statistical analysis

Statistical analyses were performed using Stata version 15.0 (StataCorp LP) and GraphPad Prism version 8.00 (GraphPad Software). Results with normal distributions were confirmed by normal distribution test and were presented as mean (standard deviation, SD) values. The association between categorical variables was examined using the Fisher exact test or the chi‐squared test, and the association between continuous variables was determined using the Mann–Whitney *U*‐test. Overall survival (OS) was calculated from the date of initial diagnosis to the date of death or last follow‐up. Progression‐free survival (PFS) was calculated from the date of initial diagnosis to the date of progression or last follow‐up. Survival was analyzed using the Kaplan–Meier method and the statistical significance was compared by the log‐rank test. The Cox proportional hazards model was used for univariate and multivariate analyses and hazard ratios (HR) and 95% confidence intervals (CI) were calculated. *p* < 0.05 was considered statistically significant.

## 3 RESULTS

### 3.1 Patient characteristics and laboratory findings

From a cohort of 217 patients with plasma cell neoplasm that underwent NGS mutation profiling, we identified 68 (31.3%) patients who had mutations in RAS signaling pathway genes (*KRAS*, *NRAS* or *BRAF*) and no other mutations. The most frequently mutated gene was *KRAS* (*n* = 35, 16.1%), followed by *NRAS* (*n* = 24, 11.1%), and *BRAF* (*n* = 10, 4.6%). One patient showed concurrent mutations in *BRAF* and *NRAS*. None of the cases had *HRAS* mutation. *KRAS* mutations involved codons 12 (*n* = 12), 61 (*n* = 12), 13 (*n* = 5), 117 (*n* = 2), 18 (*n* = 1), 19 (*n* = 1), 60 (*n* = 1) and 146 (*n* = 1). *NRAS* mutations affected codons 61 (*n* = 17), 13 (*n* = 4), 12 (*n* = 2) and 64 (*n* = 1). *BRAF* mutations altered codons 600 (*n* = 4), 469 (*n* = 3), 466 (*n* = 1), 594 (*n* = 1), and also included a *p*.581_582delinsT.

The study group included 39 men and 29 women, with a median age of 63 years (range, 35–82) at time of diagnosis. There were 51 (75%) newly diagnosed patients and 17 (25%) relapsed/refractory patients. All patients had symptomatic myeloma. Among 100 patients from the same study period who did not carry any mutations, there were 79 symptomatic myeloma, 19 smoldering myeloma, and 2 MGUS. The 79 patients with symptomatic disease were used as a control group which included 58 (73%) newly diagnosed patients and 21 (27%) relapsed/refractory patients. Notably, patients in the study cohort had lower level of hemoglobin (*p* = 0.0150) and platelet count (*p* < 0.0001), higher levels of serum LDH (*p* = 0.0172) and calcium (*p* = 0.0369), and higher percentage of BM plasma cells (*p* < 0.0001) when compared with the control group. There was no statistically significant difference in demographic and other clinical parameters including age, gender, white blood cell count, serum and urine M protein, immunoglobulin isotype, and serum levels of β‐2 microglobulin, albumin, creatinine, and total protein (Table 1).

**TABLE 1:** Clinical and laboratory characteristics of patients with plasma cell myeloma according to *KRAS/NRAS/BRAF* mutation status.

Characteristics	RAS/BRAF mutation	No mutation	*p*‐value
Age (years, median, range)	63 (35–82)	62 (40–81)	0.9776
Gender (male)	39 (57.35%)	45 (56.96%)	0.6073
WBC (K/μL, mean ± SD)	6.03 ± 2.74	6.74 ± 3.52	0.1523
Hgb (g/dL, mean ± SD)	10.22 ± 2.20	11.07 ± 2.01	0.0150
Platelet (K/μL, mean ± SD)	185.70 ± 70.38	236.40 ± 76.23	<0.0001
LDH (IU/L)			
≤618	53 (77.94%)	73 (92.41%)	0.0172
>618	15 (22.06%)	6 (7.59%)	
β2M (mg/L)			
≤3.5	32 (49.23%)	46 (58.23%)	0.5417
3.5–5.5	13 (20.00%)	14 (17.72%)	
≥5.5	20 (30.77%)	19 (24.05%)	
Albumin (g/dL)			
<3.5	16 (23.88%)	9 (11.54%)	0.0764
≥3.5	51 (76.12%)	69 (88.46%)	
BUN (mg/dL)	23.38 ± 12.76	22.36 ± 15.87	0.1418
Creatinine (mg/dL)			
≤2	57 (83.82%)	66 (83.54%)	>0.9999
>2	11 (16.18%)	13 (16.46%)	
Calcium (mg/dL)			
≤10.2	49 (73.13%)	68 (87.18%)	0.0369
>10.2	18 (26.87%)	10 (12.82%)	
Total protein (g/dL)	9.102 ± 2.341	9.058 ± 1.775	0.6900
Serum M protein (g/dL)	3.025 ± 2.280	2.929 ± 2.056	0.8864
Urine M protein (g/dL)	42.30 ± 34.08	35.04 ± 35.21	0.4200
Immunoglobulin isotype			
IgA	15 (22.06%)	24 (24.00%)	0.4123
IgG	41 (60.29%)	62 (62.00%)	
IgM	0 (0%)	1 (1.00%)	
IgD	1 (1.47%)	3 (3.00%)	
Kappa	7 (10.29%)	7 (7.00%)	
Lambda	4 (5.88%)	2 (2.00%)	
ISS			
I	23 (35.38%)	43 (54.43%)	0.1019
II	21 (32.31%)	17 (21.52%)	
III	21 (32.31%)	19 (24.05%)	
R‐ISS			
I	17 (26.15%)	38 (48.10%)	0.0094
II	35 (53.85%)	35 (44.30%)	
III	13 (20.00%)	6 (7.59%)	
BM PC%	61.06 ± 23.19	45.53 ± 22.63	<0.0001
Flow			
CD56^+^	52 (81.25%)	62 (80.52%)	>0.9999
CD117^+^	39 (60.94%)	39 (50.65%)	0.2381
CD56^+^ CD117^+^	30 (46.88%)	34 (44.16%)	0.8652
Karyotype			
Diploid	28 (43.08%)	57 (75.00%)	0.0002
Simple (1, 2) abnormality	3 (4.62%)	4 (5.26%)	
Complex (≥3 abnormalities)	34 (52.30%)	15 (19.74%)	
FISH			
*CDKN2C* deletion	11/67 (16.42%)	11/78 (14.10%)	0.8173
Extra copy of *CKS1B*	36/67 (53.73%)	28/78 (35.90%)	0.0438
t(4;14)(*FGFR3::IG* *H*)	6/22 (27.27%)	3/8 (37.50%)	0.6662
t(11;14)(*CCND1::IGH*)	11/66 (16.57%)	10/79 (12.66%)	0.6365
Mono 13/*RB1* deletion	37/67 (55.22%)	35/79 (44.30%)	0.2449
Mono 17/*TP53* deletion	10/67 (14.93%)	7/79 (8.86%)	0.3028
t(14;16)(*IGH::MA*F)	4/5 (80.00%)	0/3 (0%)	0.1429
Chemotherapy	68 (100%)	79 (100%)	>0.9999
Radiation	16 (24.24%)	15 (18.99%)	0.5425
Autologous transplant	46 (67.68%)	48 (60.76%)	0.3860
Best overall response			
CR	24 (41.38%)	32 (47.06%)	0.5909
VGPR or better	38 (65.52%)	45 (66.18%)	>0.9999
PR or better	41 (70.69%)	55 (80.88%)	0.2112
MR or better	42 (72.41%)	57 (83.82%)	0.1329
No response (SD + PD)	16 (27.59%)	11 (16.18%)	0.1329
Death	26 (38.24%)	13 (16.46%)	0.0046

Abbreviations: β2M, β‐2 microglobulin; BM PC, bone marrow plasma cells; BUN, blood urea nitrogen; CR, complete response; Hgb, hemoglobin; LDH, lactate dehydrogenase; ISS, International Staging System; MR, minor response; NDMM, newly diagnosed multiple myeloma; PD, progressive disease; PR, partial response; R‐ISS, Revised ISS; SD, stable disease; VGPR, very good partial response; WBC, white blood cells.

Using ISS, the study group included 23 (36%) patients with Stage I, 21 (32%) with Stage II and 21 (32%) with Stage III disease. Staging information was not available in three patients. In the control group, 43 (54%) patients had Stage I, 17 (22%) had Stage II and 19 (24%) had Stage III disease. There was no statistically significant difference in the distribution of ISS stage between the two groups (*p* = 0.1019). When the R‐ISS was applied to the study cohort, 17 (26%) patients had Stage I, 35 (54%) had Stage II and 13 (20%) had Stage III disease, whereas in the control group 38 (48%) patients had Stage I, 35 (44%) had Stage II and 6 (8%) had Stage III disease. Patients in the study group had a significantly higher frequency in high‐risk stage than the control group (*p* = 0.0094) (Table 1).

### 3.2 Cytogenetic results

**FIGURE 1:**
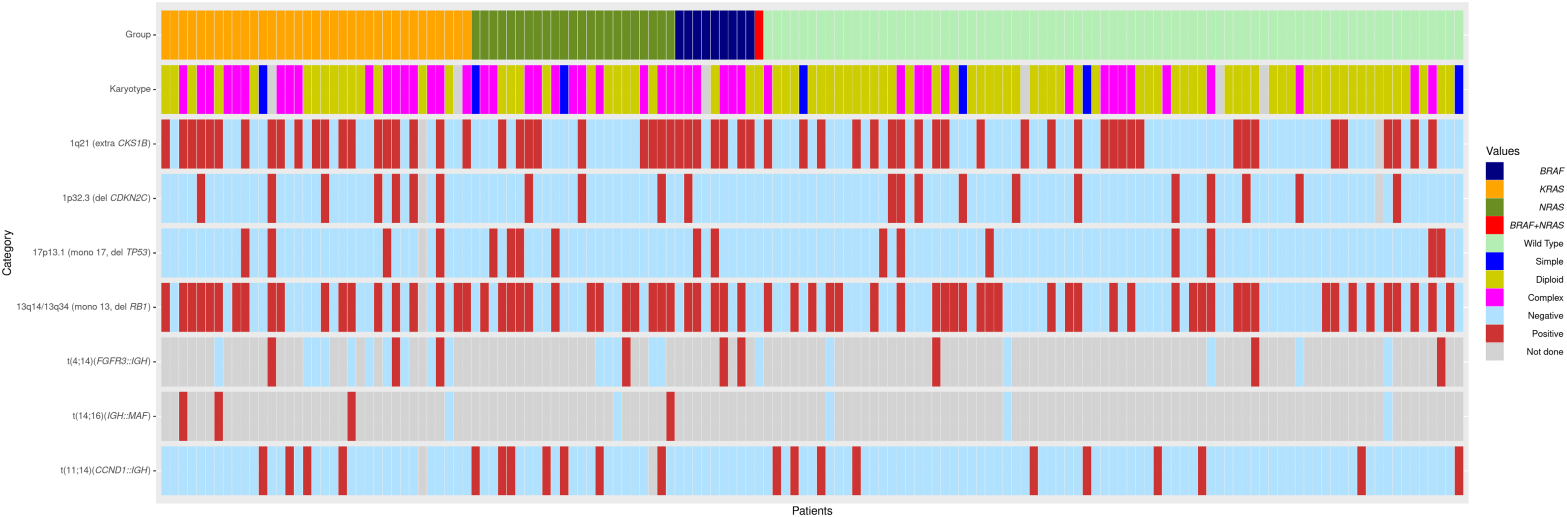
Gene mutations and cytogenetic abnormalities. The mutation status (top row), karyotype (second row), and abnormalities detected by fluorescence in situ hybridization (rows 3–9) for each individual cases are listed. Each vertical bar represents an individual patient.

In the study group, 28 (43%) patients had normal karyotype, 3 (5%) had simple abnormal karyotype with 1–2 chromosomal aberration(s) and 34 (52%) had complex karyotype (≥3 chromosomal aberrations, and at least one with structural abnormality). In contrast, in the control group, 57 (75%) patients had normal karyotype, 4 (5%) had simple chromosomal aberration(s) and 15 (20%) had complex karyotype. There were significantly more patients with complex karyotype in the study group than the control group (*p* = 0.0002) (Table 1, Figure 1). We also compared chromosomal abnormalities detected by FISH. *RAS/BRAF*‐mutated cases showed a significantly higher frequency of gain/amplification of *CKS1B* (54% vs. 36%, *p* = 0.0438). t(14;16)(*IGH::MAF*) was identified in 4 patients in the study group, but none in the control group. There were no significant differences between the two groups in the frequencies of *CDKN2C* deletion, t(4;14)(*FGFR3*::*IGH*), t(11;14)(*CCND1*::*IGH*), monosomy 13/*RB1* deletion, and deletion of *TP53* or monosomy 17 (Table 1, Figure 1).

### 3.3 Clinical outcome and prognostic factors

All patients received standard clinical management with immunomodulatory drugs and/or proteasome inhibitors. Sixteen (24%) patients in the study group and 15 (19%) in the control group received radiation therapy for bone lesions. In the study group, 46 (68%) and 1 (1%) patient additionally underwent autologous and allogeneic stem cell transplantation (SCT), respectively. In the control group, 48 (61%) additionally received autologous SCT, and none received allogeneic SCT. Data on best overall responses to therapy were available for 58 and 68 patients in the study and control groups, respectively. Among these patients, 24 (41%) from the study group and 32 (47%) from the control group achieved complete remission, and 41 (71%) from the study group and 55 (81%) from the control group achieved at least partial response. There was no statistically significant difference in the response rate between the two groups (Table 1).

**FIGURE 2:**
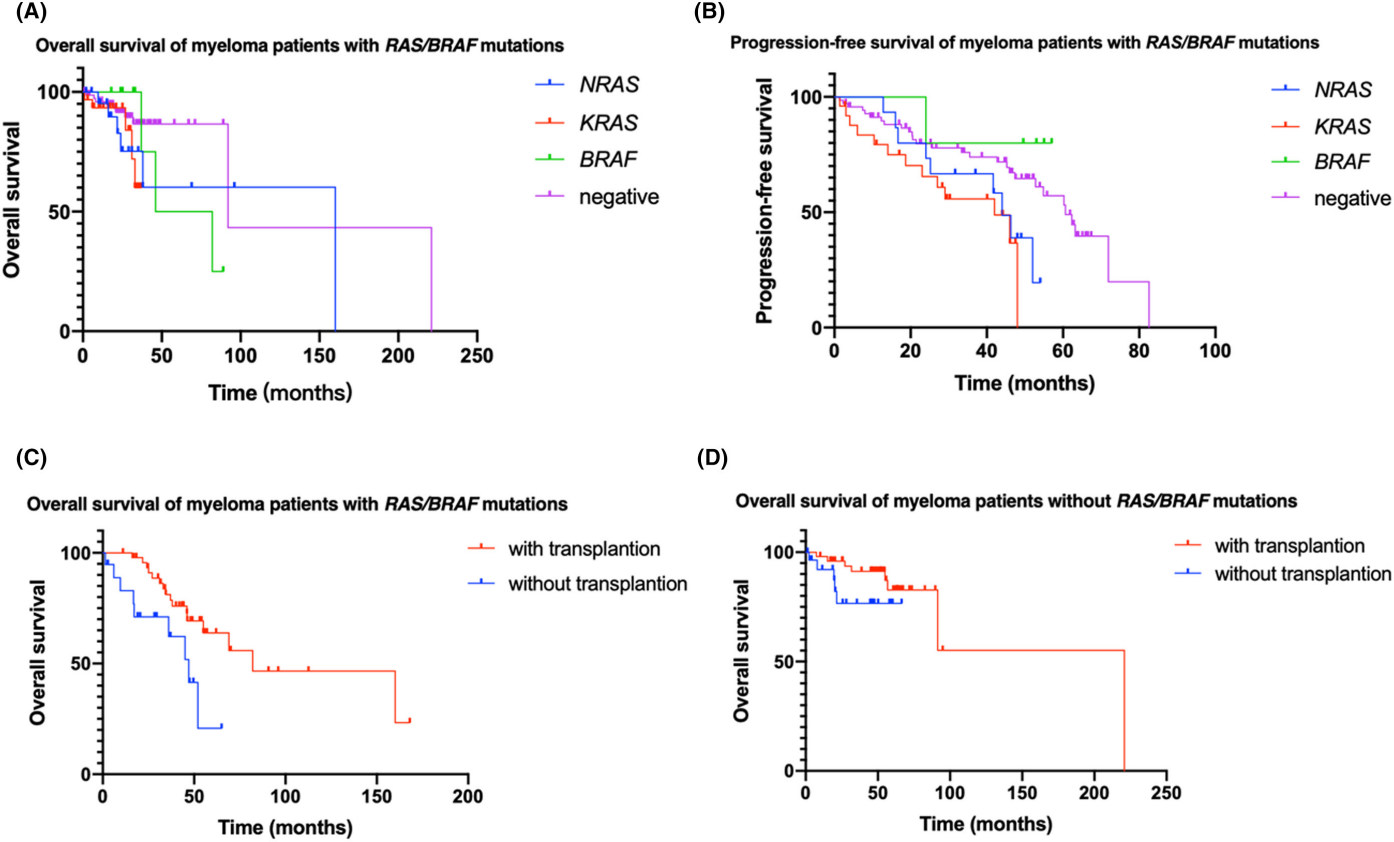
Overall survival and progression‐free survival of myeloma patients based on (A and B) *KRAS/NRAS/BRAF* mutation status and (C and D) autologous stem cell transplantation.

With a median follow up of 42 months (range, 1.4–168.1) and 49 months (range, 0.3–276.6) for the study and control groups, respectively, 26 (38%) patients in the study group and 13 (16%) patients in control group died (*p* = 0.046, Table 1). The median OS was also significantly shorter for patients in the study group (69 months) than patients in the control group (220.7 months, *p* = 0.0023). Further analysis showed that the median OS was 55, 69, and 82 months for patients who carried *NRAS*, *KRAS*, and *BRAF* mutations alone, respectively. Mutations in *KRAS* and *NRAS*, but not *BRAF*, were associated with significantly shorter OS, compared with the control group (*p* = 0.0015 and *p* = 0.0415, respectively) (Figure 2A). Moreover, the median PFS was significantly shorter for patients in the study group (46.0 months) than patients in the control group (60.6 months, *p* = 0.0311). Further analysis showed that the PFS was 42 months for patients with *KRAS* mutations and 44 months for patients with *NRAS* mutations, and that only patients with *KRAS* mutations had significantly shorter PFS compared with the control group (*p* = 0.0105) (Figure 2B). In the study group, patients who received SCT showed a survival advantage over patients who did not receive SCT (median OS: 82 vs. 47 months, *p* = 0.0121; median PFS: 46.3 vs. 29.0 months, *p* = 0.1514) (Figure 2C). In the control group, whereas patients who received SCT had longer OS and PFS than those who did not receive SCT, this did not reach statistical significance, likely due to the relatively small sample size (Figure 2D). There was no significant difference in OS between patients who received and did not receive radiation therapy in each group.

Univariate analysis showed that *KRAS* mutation, *NRAS* mutation, lower hemoglobin count, elevated LDH level, higher R‐ISS stage, complex karyotype, gain/amplification of *CKS1B*, monosomy 13/*RB1* deletion, and lack of autologous SCT were associated with poorer prognosis (Table 2). Multivariate analysis confirmed that *KRAS* mutation had an independent negative impact on OS (*p* = 0.045; HR: 7.400; 95% CI: 1.042–52.560). Other independent negative prognostic factors in the multivariate analysis included lower hemoglobin count, high serum calcium level, higher ISS stage, and lack of autologous SCT (Table 3).

**TABLE 2:** Univariate analysis of overall survival in patients with plasma cell myeloma.

Variable	No. of patients	No. of deaths	Median OS	*p*‐value
*KRAS* mutation	35	13	69	0.0015
Wild type	79	13	220.7	
*NRAS* mutation	23	9	55	0.0415
Wild type	79	13	220.7	
*BRAF* mutation	9	4	82	0.2456
Wild type	79	13	220.7	
Age at diagnosis				
≥65	63	17	69	0.3145
<65	84	22	160	
Gender				
Female	63	17	82	0.6841
Male	84	22	160	
Hemoglobin				
<10 g/dL	58	21	69	0.0362
≥10 g/dL	89	18	220.7	
Lactate dehydrogenase				
≤618 IU/L	126	26	220.7	0.0002
>618 IU/L	21	13	38	
β‐2 microglobulin				
<3.5 mg/L	78	16	160	0.5845
3.5–5.5 mg/L	28	10	69	
≥5.5 mg/L	39	12	91.5	
Albumin				
<3.5 g/dL	24	10	54.87	0.0929
≥3.5 g/dL	121	28	91.50	
Creatinine				
≤2 mg/dL	123	35	91.5	0.4983
>2 mg/dL	24	4	Undefined	
Calcium				
≤10.2 mg/dL	117	29	91.5	0.1011
>10.2 mg/dL	28	10	Undefined	
Heavy chain				
IgA	33	11	160	0.5189
IgG	90	22	91.5	
IgM	1	0	Undefined	
IgD	4	1	Undefined	
Light chain	18	5	82	
Light chain				
Κ	86	20	160	0.5351
λ	61	19	91.5	
Isotype				
Heavy chain	129	34	160	0.3159
Light chain	18	5	82	
ISS stage				
I	65	11	160	0.2424
II	39	15	69	
III	40	12	220.7	
R‐ISS stage				
I	55	7	Undefined	0.0118
II	70	23	91.5	
III	19	8	47	
Bone marrow plasma cells				
<60%	74	13	220.7	0.6008
≥60%	73	12	91.5	
Flow			P/N	
CD56^+^	114 (80.85%)	26 (74.29%)	160/82	0.2511
CD117^+^	78 (55.32%)	16 (45.71%)	Undefined/160	0.5147
CD56^+^ and CD117^+^	64 (45.29%)	11 (31.43%)	Undefined/160	0.1681
Karyotype				
Diploid	85	16	Undefined	0.0234
Simple^1, 2^	7	0	Undefined	
Complex (≥3)	49	20	69	
FISH aberrations			P/N	
*CDKN2C* deletion	22 (15.17%)	8 (21.62%)	Undefined/160	0.2915
Extra copy of *CKS1B*	64 (44.14%)	24 (64.86%)	69/220.7	0.0041
t(4;14)(*FGFR3::IGH*)	8 (28.57%)	5 (45.45%)	46.2/Undefined	0.5775
t(11;14)(*CCND1::IGH*)	21 (14.48%)	4 (10.81%)	Undefined/91.5	0.4571
Mono 13/*RB1* deletion	72 (49.32%)	24 (63.16%)	82/160	0.0405
Mono 17/*TP53* deletion	17 (11.64%)	6 (15.80%)	82/160	0.5438
t(14;16)(*IGH::MAF*)	4 (50%)	2 (50%)	45/37.1	0.9229
Autologous transplantation				
Yes	97	24	160	0.0056
No	48	14	52	
Extramedullary disease				
Yes	31	11	220.7	0.5415
No	114	27	91.5	

Abbreviations: ISS, International Staging System; OS, overall survival; P/N, positive/negative; R‐ISS, Revised ISS.

**TABLE 3:** Multivariate analysis of overall survival in patients with plasma cell myeloma.

Variable	*p*‐value	HR (95% CI)
*KRAS* mutation	0.045	7.40 (1.04–52.56)
Hemoglobin	0.027	0.14 (0.02–0.79)
Serum calcium level	0.004	12.37 (2.27–67.31)
ISS stage	0.020	0.12 (0.02–0.72)
Autologous transplantation	0.005	0.09 (0.02–0.50)

## 4 DISCUSSION

The RAS/RAF/MEK/ERK signaling pathway plays an important role in many fundamental biological processes, including cell proliferation, apoptosis, adhesion, migration and angiogenesis, and has been reported to be activated in about half of myeloma cases.^25^, ^26^, ^27^, ^28^ Activating mutations in the RAS gene family have been identified in 20–50% of newly diagnosed myeloma and 45%–80% of relapsed/refractory myeloma cases.^12^, ^19^, ^25^ These mutations are believed to contribute to disease progression, from MGUS to myeloma.^6^ However, studies on the impact of RAS pathway mutations on survival have led to contradictory results. To explore the clinical and biological significance of *RAS/BRAF* mutations in patients with myeloma, we studied the association of *RAS/BRAF* mutations with a panel of parameters including laboratory data, cytogenetic aberrations, responses to therapy, and clinical outcome. RAS pathway mutations, including *KRAS*, *NRAS*, and *BRAF*, were detected in about a third of patients in this series. Whereas most mutations affected codons 61, 12, and 13 of *KRAS/NRAS* and codon 600 of *BRAF*, we also detected point mutations in many other codons as well as a frameshift *BRAF* mutation. *KRAS* and *NRAS* mutations were mutually exclusive in our series, but one patient carried concurrent mutations in *NRAS* and *BRAF*. None of the patients with *RAS/BRAF* mutations in this cohort had MGUS or smoldering myeloma, supporting the idea that RAS pathway mutation is associated with symptomatic myeloma.

It has been reported that *KRAS* and *NRAS* mutations are associated with adverse clinical features accounting for more aggressive clinical course. These features include higher tumor burden, advanced ISS stage (II and III), lower hemoglobin, and more frequent lytic bone lesions.^19^ However, in other studies activating RAS mutations did not correlate with the clinical stage.^18^, ^29^ In keeping with the findings of Chng and colleagues,^19^ patients in this study group had lower hemoglobin and platelet counts, higher levels of serum LDH and calcium, higher BM tumor burden and higher frequency of advanced‐stage disease than the control group. In addition, more patients in the *RAS/BRAF*‐mutated group had complex karyotype, gain/amplification of *CKS1B* and t(14;16)(*IGH::MAF*) (although the latter is not statistically significant due to the small number of patients). The R‐ISS incorporates serum LDH level and chromosomal abnormalities detected by FISH because these elements define biologic features of myeloma.^8^, ^30^ In the R‐ISS, high‐risk disease is characterized by the presence of del(17p), translocation t(4;14) and/or translocation t(14;16)(q32;q23).^8^ The higher serum LDH level and more patients carrying t(14;16) in the study cohort may contribute to the enriched number of higher R‐ISS patients.

In this case series, the median OS and PFS were significantly shorter for patients in the *RAS/BRAF*‐mutated group versus patients in the control group. When comparing each individual mutation to wild type, we found that *KRAS* and *NRAS* mutations, but not *BRAF* mutation were associated with significantly shorter OS, and only *KRAS* mutation was associated with shorter PFS. Furthermore, multivariate analysis showed that only *KRAS* mutation was proven to be an independent prognostic factor. These results are in keeping with some earlier studies in which *KRAS* mutation had the most significant impact on the survival of myeloma patients.^18^, ^19^, ^20^, ^21^ We noted conflicting reports regarding the prognostic impact of RAS mutation. Mulligan et al. showed that *NRAS*, but not *KRAS* mutation, had a negative impact on the clinical outcome of patients treated with bortezomib.^9^ Walker et al. reported that *RAS* mutation had no prognostic value.^13^ Gebauer et al. found that *RAS* mutations had a favorable prognostic impact in myeloma patients treated with high‐dose melphalan and autologous SCT.^22^ These discrepant results, in part, may be explained by different patient populations, treatment regimens, response criteria, and presence of other molecular genetic aberrations. Cheung et al. demonstrated that *BRAF* V600E mutation was significantly associated with hypercalcemia and conferred inferior patient survival in younger, but not elderly myeloma patients.^17^ We did not observe any survival impact of *BRAF* mutation, which may be due to the fact that the number of *BRAF*‐mutated (in particularly *BRAF* V600E) patients was small, our patient population is composed of adults and that patients may have received different therapeutic regimens.

*RAS/BRAF* mutations in myeloma offer a potential opportunity to use a targeted approach with MAPK–ERK inhibitors. Vemurafenib, a BRAF inhibitor, has shown promise in the treatment of patients with metastatic melanoma, non‐small cell lung cancer, papillary thyroid carcinoma, hairy cell leukemia, and Langerhans cell histiocytosis.^31^, ^32^, ^33^, ^34^, ^35^ However, vemurafenib as a single agent in the treatment of *BRAF*‐mutated myeloma patients has resulted in a low response rate and early resistance.^36^ Subsequently, Ernst et al reported successful treatment with a combination of the BRAF inhibitor dabrafenib and the MEK inhibitor trametinib in a patient with *BRAF* V600E‐mutated refractory myeloma.^37^ Although direct pharmacological targeting of mutated *RAS* has not led to widespread clinical therapies at this time, Downward et al. suggested that mutant *KRAS* rendered tumor cells more susceptible to proteasome inhibitors.^38^ Furthermore, the association between *RAS/BRAF* mutations and complex karyotypes suggests that this group of patients may benefit from checkpoint inhibitors.^39^

In summary, we show that gene mutations involving the RAS pathway occurring in about one third of myeloma cases, and are hallmark of aggressive disease. In this cohort, these mutations were associated with a complex karyotype, advanced stage disease and shorter PFS and OS. The association of *RAS/RAF* mutations with adverse biological variables suggests that analysis of these genes should be included in routine NGS‐based mutation panels for the clinical work‐up of myeloma patients. Further studies to explore the potential therapeutic effects of RAS/BRAF/MAPK inhibitors in this patient subset are needed.

## AUTHOR CONTRIBUTIONS

**Nianyi Li:** Conceptualization (supporting); data curation (lead); formal analysis (lead); writing – original draft (equal); writing – review and editing (supporting). **Lin Pei:** Conceptualization (supporting); data curation (supporting); writing – review and editing (supporting). **Zhuang Zuo:** Data curation (supporting); writing – review and editing (supporting). **M. James You:** Data curation (supporting); writing – review and editing (supporting). **Wen Shuai:** Data curation (supporting); writing – review and editing (supporting). **Robert Orlowski:** Data curation (supporting); writing – review and editing (supporting). **Elisabet Manasanch:** Data curation (supporting); writing – review and editing (supporting). **Shaoying Li:** Data curation (supporting); writing – review and editing (supporting). **Jie Xu:** Data curation (supporting); writing – review and editing (supporting). **Sofia Garces:** Data curation (supporting); writing – review and editing (supporting). **Fatima Zahra Jelloul:** Data curation (supporting); writing – review and editing (supporting). **Zhenya Tang:** Data curation (supporting); writing – review and editing (supporting). **Wei Wang:** Data curation (supporting); writing – review and editing (supporting). **L.Jefferey Medeiros:** Data curation (supporting); writing – review and editing (supporting). **C. Cameron Yin:** Conceptualization (lead); data curation (equal); formal analysis (equal); supervision (lead); writing – original draft (equal); writing – review and editing (lead).

## FUNDING INFORMATION

The authors received no specific funding for this work.

## CONFLICT OF INTEREST STATEMENT

The authors disclose no conflict of interests.

## INSTITUTIONAL REVIEW BOARD STATEMENT

This study was conducted in accordance with the Declaration of Helsinki and approved by the Institutional Review Board of the University of Texas MD Anderson Cancer Center (PA14‐0024 Study of Molecular Genetic Abnormalities and Its Association with Clinicopathologic Features in Patients with Lymphomas and Myelomas).

## Data Availability

All data generated or analyzed during this study are included in this published article.
